# Sociodemographic inequalities in the trends of different types of leisure-time physical activity among Brazilian adults between 2006 and 2019

**DOI:** 10.1186/s12939-022-01728-y

**Published:** 2022-08-29

**Authors:** Raphael H. O. Araujo, André O. Werneck, Danilo R. Silva, Gilmar M. Jesus

**Affiliations:** 1 Graduation Program in Health Sciences, Londrina State University (UEL), Londrina, Brazil; 2 Center for Epidemiological Research in Nutrition and Health, Department of Nutrition, School of Public Health, University of São Paulo (USP), São Paulo, Brazil; 3 Department of Physical Education, Federal University of Sergipe (UFS), São Cristóvão, Brazil; 4 Department of Sports and Computer Science, Universidad Pablo de Olavide (UPO), Seville, Spain; 5 Faculty of Health Sciences, Universidad Autónoma de Chile, Santiago, Chile; 6 Department of Health, State University of Feira de Santana (UEFS), Feira de Santana, Bahia Brazil

**Keywords:** Physical activity, Inequality, Gender, Ethnicity

## Abstract

**Background:**

The current study aimed to describe the trends in gender, ethnicity, and education inequalities of types of leisure-time physical activity (LTPA) practiced by Brazilian adults from 2006 to 2019.

**Methods:**

We used data from 2006 to 2019 of the Brazilian Surveillance System for Risk and Protective Factors for Chronic Diseases by Telephone Survey, which is an annual survey with a representative sample of adults (≥ 18y) living in state capital cities. The types of LTPA considered were walking, running, strength/gymnastics, sports, other LTPA, and no LTPA participation. Gender (women or men), ethnicity (white, black, brown, or yellow/indigenous), and years of formal education were also self-reported. We used relative frequencies and their respective 95% confidence intervals to analyze trends. The absolute and relative differences between the proportions were used to assess the inequalities.

**Results:**

We observed increases in inequalities related to gender and education (running and strength/gymnastics), while gender inequalities for sports, other types, and no LTPA participation decreased. There were persistent inequalities related to gender (walking) and education (sports, other types, and no LTPA participation). Considering ethnicity, we noted increases in inequality for strength/gymnastics, where white adults were more active than black and brown adults. In addition, white adults reported more access to LTPA than brown adults over the years analyzed.

**Conclusion:**

Women, black and brown people, and subjects with less schooling were the most unfavored groups. While some inequalities persisted over the years, others increased, such as ethnicity and education inequalities for strength/gymnastics.

**Supplementary Information:**

The online version contains supplementary material available at 10.1186/s12939-022-01728-y.

## Background

Regular physical activity is associated with several health benefits, such as lower risk of hypertension, reduced risk of depression, and improved cognition [1–3]. Among the physical activity domains, leisure-time physical activity (LTPA) is a well-established protective factor for several negative health outcomes, including mental disorders [4], different types of cancer [5], mortality due to cardiovascular disease, and all-cause mortality [6]. Beyond its association with health outcomes, LTPA is also associated with a higher quality of life, and wellbeing [7]. However, the promotion of LTPA remains a global challenge. In Brazil, a study carried out with adults living in capital cities reported that the prevalence of LTPA participation (at least one day per week) was 44% in 2006 and 54% in 2016 [8]. The most widely reported types of LTPA practiced in Brazil were outdoor walking, soccer, and strength training [9]. However, there were important inequalities related to gender, age, and income in the different types of physical activity [9].

Given the inequalities in LTPA, continuous monitoring of these indicators is essential to provide evidence on the trends in inequality and to plan public policies to target risk groups. In Brazil, both the Brazilian Surveillance System for Risk and Protective Factors for Chronic Diseases by Telephone Survey (VIGITEL) and the National Health Survey (PNS) provide monitoring data on LTPA in the adult population (≥ 18 years). However, most previous studies carried out in Brazil analyzed only the trend in the level of LTPA [8, 10–12], with little available information about trends in types of LTPA [13, 14]. Information on the most commonly practiced types of physical activity is strategic because the type of physical activity provides quick, practical, and objective information on preferences and contexts, aspects influenced by culture, the development of personal skills, and inequalities in access to facilities and equipment suitable for practice.

Although previous studies investigated the trends in different types of LTPA [15] and social inequalities in the participation in different types of LTPA [9], the trends in inequalities in the participation in different types of LTPA were not explored [15]. Monitoring these trends makes it important to analyze how the participation in LTPA is variating among populational groups constantly unfavored (e.g., women and low-income people) [9], as well as to identify whether changes in the participation in different types of LTPA have been driven by specific subgroups. In addition, given that studies on trends in LTPA level have shown increases, especially among the richest groups [16], increases may occur in specific activities, such as activities traditionally paid. Consequently, understanding these trends can support public policies to reduce inequalities in access to LTPA. Thus, the current study aimed to describe the trends in gender, ethnicity, and education inequalities of types of LTPA practiced by Brazilian adults from 2006 to 2019.

## Methods

### Design and sample

This study used data from 2006 to 2019 from the VIGITEL survey (*Brazilian Surveillance System for Risk and Protective Factors for Chronic Diseases by Telephone Survey*). The VIGITEL is an annual telephone-based cross-sectional survey with adults (≥ 18 years old) living in Brazilian state capitals (26 capitals) and the Federal District. To estimate the frequencies of risk factors among the population [95% confidence interval (CI) and maximum error of 2%)], the minimum sample size in each city was 2,000 participants. Initially, 5,000 telephone lines were drawn in each city, with stratification by region. After the exclusion of commercial and non-operational lines, 2,000 lines were randomly selected, and in each household, one adult was randomly selected to respond to the questionnaire. Further information is available elsewhere [17].

### Leisure-time physical activity

LTPA was assessed by the question: “In the last three months, did you practice any type of exercise or sports?”. Possible answers were “yes” or “no”. Those who answered yes, responded to the following question “What is the main type of physical exercise or sport that you practiced?”. Possible answers were categorized into walking (walking and treadmill walking), running (running and treadmill running), strength/gymnastics (strength training; gym aerobics; water aerobics; gymnastics), sports (swimming; martial arts; football; basketball; volleyball; and tennis), and other LTPA (outdoor or indoor cycling; dance [ballet, belly dance, ballroom dance]; or other).

### Socio-demographic characteristics

The socio-demographic characteristics consisted of gender (men and women), ethnicity (white, black, brown, and yellow/indigenous), and education (0–8, 9–11, and 12 + years of schooling).

### Statistics

The descriptive analysis was performed by relative frequencies and their respective 95% confidence intervals (95%CI). The 95%CI were used to identify the trends in the prevalence of different types of leisure-time physical activity between 2006 and 2019. Absolute differences were used to present the gender and ethnic inequalities, with results reported in percentage points (p.p.). The Slope Index of Inequality (SII) was used to measure the education inequality considering the intermediate group (9–11 years of schooling). Equiplots were created using the “equiplot” command (https://equidade.org/equiplot). The SII was calculated using the “siilogit” command (https://equidade.org/ineq-measures). All the analyses considered the sampling weights. The analyses were conducted in Stata 15.0 software.

## Results

**Fig. 1:**
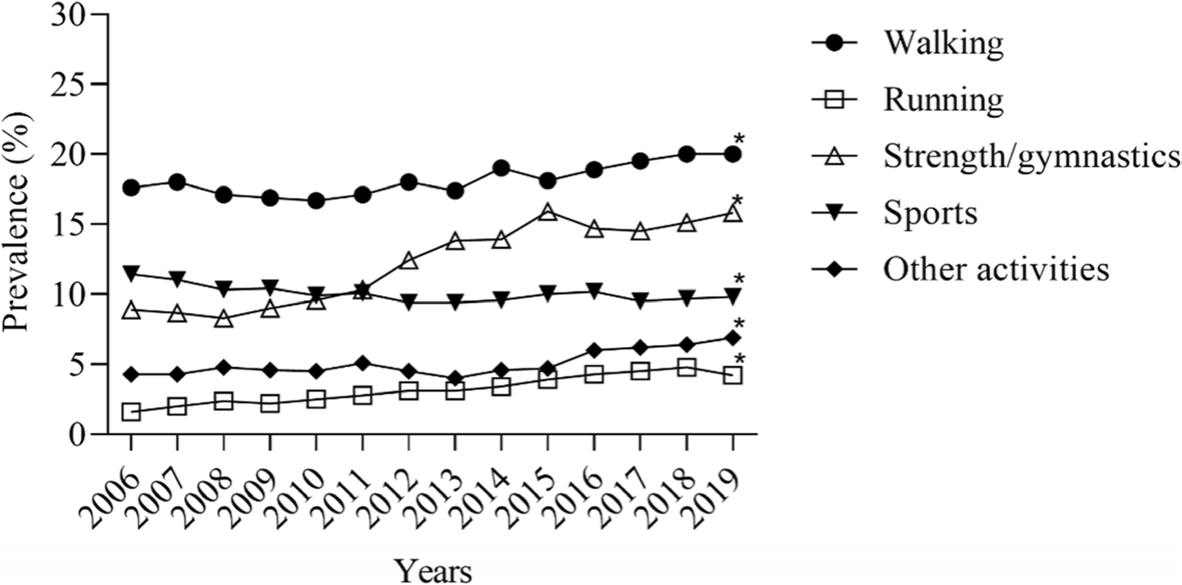
Trends in the prevalence of different types of leisure-time physical activity among Brazilian adults living in capital cities. *There is no overlap of 95% confidence intervals between 2006 and 2019

Figure 1 presents the trends in walking, running, strength/gymnastics, sports, other types of LTPA, and no LTPA participation between 2006 and 2019. We noticed increases in participation in walking, running, strength/gymnastics, and other activities. On the other hand, there was a decrease in sports participation. In addition, we observed a reduction in the frequencies of those who did not participate in LTPA between 2006 [55.9% (95% CI, 55.0; 56.7)] and 2019 [43.0% (95% CI, 42.0; 44.0)].

**Fig. 2:**
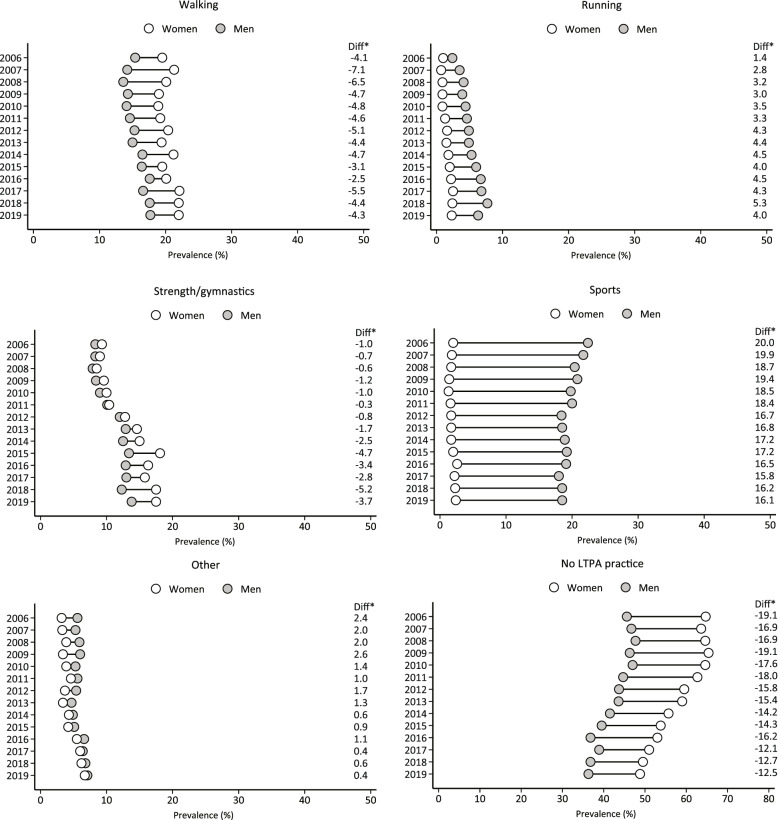
Trends in the prevalence of different types of LTPA and no LTPA participation among Brazilian adults living in capital cities, according to gender. Note: Diff*, the absolute difference in percentage points (men versus women). LTPA, leisure-time physical activity

Figure 2 shows the trends in different types of LTPA and no LTPA participation, between 2006 and 2019, according to gender. In general, we observed an increase in the percentage of women and men who participated in walking [(women, 2006: 19.5% vs. 2019: 22.0%); (men, 2006: 15.4% vs. 2019: 17.7%)], running [(women, 2006: 1.0% vs. 2019: 2.3%); (men, 2006: 2.4% vs. 2019: 6.3%)], and strength/gymnastics [(women, 2006: 9.3% vs. 2019: 17.5%); (men, 2006: 8.3% vs. 2019: 13.8%)]. There was a decrease in sports participation among men (2006: 22.4% vs. 2019: 18.5%), while no changes were noted among women (2006: 2.0% vs. 2019: 2.4%). In addition, we observed a slight decrease in the gender disparity in sports participation (the type of LTPA with the highest gender disparity), with a difference between men and women of 20.4p.p. in 2006 versus 16.1p.p. in 2019. There was a decrease in the prevalence of no LTPA participation among women (2006: 64.7% vs. 2019: 48.8%) and men (2006: 45.6% vs. 2019: 36.3%), however, the prevalence of women who reported no LTPA participation in 2019 (48.8%) was still higher than among men in 2006 (45.6%).

**Fig. 3:**
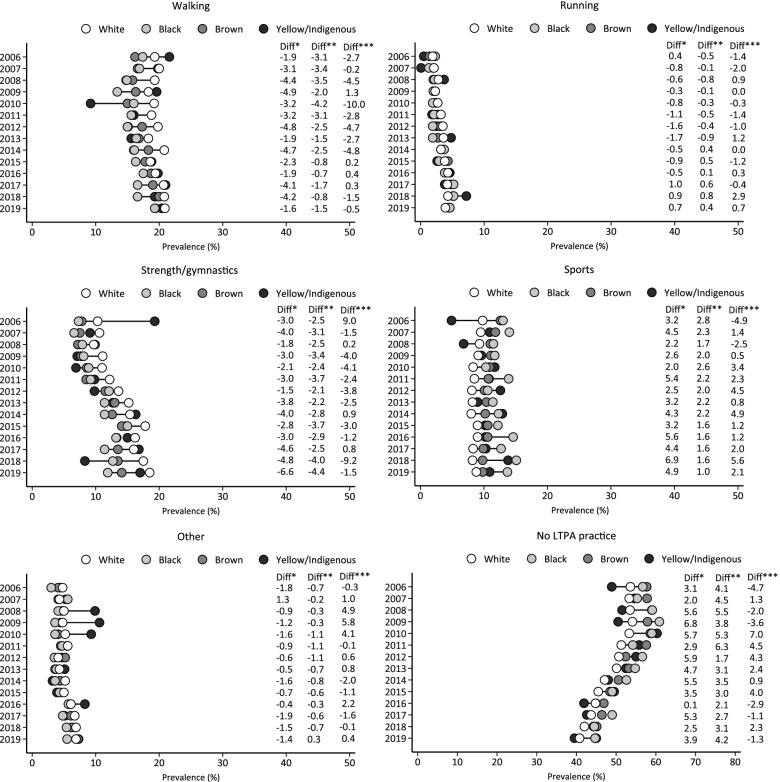
Trends in the prevalence of different types of LTPA and no LTPA participation among Brazilian adults living in capital cities, according to ethnicity. Note: Diff is referring to the absolute difference in percentage points. Diff*, black versus white. Diff**, brown versus white. Diff***, Yellow/Indigenous versus white. LTPA, leisure-time physical activity

Figure 3 shows the trends in different types of LTPA and no LTPA participation, between 2006 and 2019, according to ethnicity. There was an increase in the prevalence of walking among brown (2006: 16.2% vs. 2019: 19.4%), running among white (2006: 1.9% vs. 2019: 3.9%), brown (2006: 1.4% vs. 2019: 4.3%), and yellow/indigenous adults (2006: 0.5% vs. 2019: 4.6%), strength/gymnastics among white (2006: 10.3% vs. 2019: 18.5), black (2006: 7.3% vs. 2019: 11.9%), and brown (2006: 7.8% vs. 2019: 14.1%), and other types of LTPA among white (2006: 4.8% vs. 2019: 6.9%) and brown (2006: 4.1% vs. 2019: 7.2%). There was a decrease in sports participation among brown adults (2006: 12.6% vs. 2019: 9.8%). In addition, the prevalence of no LTPA participation decreased among white (2006: 53.6% vs. 2019: 40.8%), black (2006: 56.7% vs. 2019: 44.7%) and brown (2006: 57.7% vs. 2019: 45.0%). Concerning the disparities, in 2006, white ethnicities reported more participation in strength/gymnastics activities than black and brown ethnicities (black versus white: -3.0p.p.; brown versus white: -2.5p.p.); in addition, we observed increases in absolute differences over the years analyzed (2019, black versus white, -6.6p.p.; brown versus white, -4.4p.p.). In 2006, there was no difference in sports participation between black and white and between black and brown. However, in 2019, black adults (13.7%) presented more participation in sports than white (8.8%) and brown (9.8%).

**Fig. 4:**
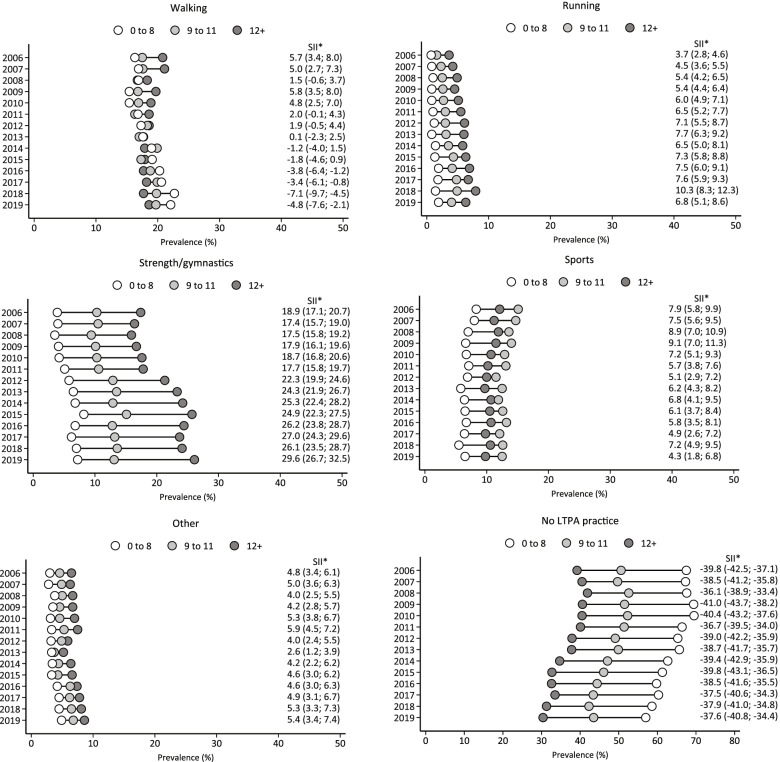
Trends in the prevalence of different types of LTPA and no LTPA participation among Brazilian adults living in capital cities, according to education level. Note: SII, Slope Index of Inequality. LTPA, leisure-time physical activity

Figure 4 shows the trends in different types of LTPA and no LTPA participation, between 2006 and 2019, according to education level. We observed increases in running (0 to 8 years, 2006: 0.7% vs. 2019: 1.9%; 9 to 11 years, 2006: 1.6% vs. 2019: 4.0%; 12 + years, 2006: 3.6% vs. 2019: 6.3%), strength/gymnastic (0 to 8 years, 2006: 3.9% vs. 2019: 7.2%; 9 to 11 years, 2006: 10.3% vs. 2019: 13.1%; 12 + years, 2006: 17.4% vs. 2019: 26.1%), and other types of LTPA (0 to 8 years, 2006: 3.0% vs. 2019: 4.9%; 9 to 11 years, 2006: 4.6% vs. 2019: 6.8%; 12 + years, 2006: 6.5% vs. 2019: 8.6%) among all education achievement categories. There was an increase in the prevalence of walking among those with 0 to 8 years of schooling (2006: 16.3% vs. 2019 22.1%) and 9 to 11 years of schooling (2006: 17.5% vs. 2019: 19.7%), while no change was noted among those with 12 + years of schooling. Sports participation decreased among those with 9 to 11 years of schooling (2006: 15.1% vs. 2019: 12.5%). The main disparities in LTPA analyzed were observed in activities of strength/gymnastics, whose SII ranged from 18.9 (95%CI: 17.1; 20.7) to 29.6 (95%CI: 26.7; 32.5). Although the prevalence of no LTPA participation decreased in all categories of education level [(0 to 8 years, 2006: 67.5% vs. 2019: 57.0%); (9 to 11 years, 2006: 50.7% vs. 2019: 43.5%); (12 + years, 2006: 39.2% vs. 2019: 30.4%)], the inequality between the groups remained constant over the years [SII, 2006: -39.8 (95%CI: -42.5; -37.1); SII, 2019: -37.6 (-40.8; -34.4)]. Furthermore, the prevalence of no LTPA participation among those with 0 to 8 and 9 to 11 years of schooling in 2019 (57.0% and 43.5%, respectively) was still higher than among those with 12 + years of schooling in 2006 (39.2%).

The Supplementary Tables 1, 2 and 3 present the prevalence and their respective 95% CI of walking, running, strength/gymnastics, sports, other types of LTPA, and no LTPA participation between 2006 and 2019, according to gender, ethnicity, and education level, respectively.

## Discussion

We aimed to analyze the trends in the gender, ethnicity, and education inequalities of different types of LTPA among adults living in Brazilian capitals. Despite the general increase in LTPA participation over the years, focusing only on the overall prevalence can hide inequalities among population subgroups. In this sense, our stratified analysis revealed persistent disparities related to gender (walking, running, sports, and no LTPA participation), ethnicity (strength/gymnastics and no LTPA participation), and education (running, strength/gymnastics, sports, other types of LTPA, and no LTPA participation).

Several studies have reported gender inequality in physical activity as a major challenge in physical activity promotion [18, 19]. Some factors that help explain these inequalities have been reported in the literature, such as social and cultural norms where involvement in household chores is much higher among women than among men. Even among youth, studies have demonstrated that parental and school support for participation in sports is higher for boys than for girls, demonstrating that actions aiming to tackle gender inequality need to start early [20]. In Brazil, Cruz et al. [8] observed that between 2006 and 2016, men were more active during leisure time than women, with a slight decrease in gender inequality. However, we observed different trends depending on the type of LTPA, with an increasing trend of inequality in women being more engaged in walking and strength/gymnastics, and an increasing trend in men being more engaged in running than women. Despite the changes in the inequalities, we highlight that there was persistent gender inequality, especially in sports participation, which could be the main driver for a higher prevalence of no LTPA participation among women. Although men and women can prefer certain types of physical activities, men reported more LTPA participation than women over all the years analyzed. Thus, our findings suggest that differences in sports participation can be not only a preference, but also a difference in access and support. In this way, promoting the participation of women in sports is a key point to decreasing the gender inequality in LTPA, as well as increasing physical activity levels worldwide [21, 22].

We noted trends in the ethnicity inequalities, especially for strength/gymnastics. There were also consistent inequalities over the years, where white people had more access to these activities than their black and brown peers, and the proportion of no LTPA participation was greater among brown than white adults over the years analyzed. The association between ethnicity and LTPA among adults has already been presented in the Brazilian context [23, 24], but less is known about the different types of LTPA [9]. Therefore, our findings can be interpreted considering different perspectives. In Brazil, there is great inequality in society concerning ethnicity, including structural racism. For several historical reasons, poverty levels are higher among brown and black people. For example, the percentage of white people with an income below 5.5 dollars a day is 15.4%, while the percentage of black/ brown ethnicities is 32.9% [25]. Higher poverty rates can lead to less access to private structures for physical activity, such as gyms. These findings demonstrate the urgent need for public policies to improve income distribution, giving greater autonomy for individuals to engage in LTPA, as well as enhance other health conditions [26, 27].

Our results also indicated that education inequalities increased over the years for strength/gymnastics, with increased participation among those with a higher education level than those with a lower education level. The inequalities also persisted over time for the other types of LTPA, where those with more education presented a higher prevalence of the different LTPA types, except for walking. Likewise, although LTPA is increasing among Brazilian adults, this was especially observed among the higher education group [16]. Thus, our findings expand previous research by showing that the biggest inequalities occurred in strength/gymnastics activities. Some facts can help in explaining these inequalities, for instance, “strength/gymnastics” activities commonly include membership fees, and the fact that individuals with higher education may have higher incomes than those with less education increases the probability of access to this type of LTPA [28]. Other reasons could also partly explain the maintenance of these inequalities over time, such as the unequal distribution of physical activity facilities, which especially affects areas with disadvantaged socioeconomic backgrounds [29]. Our results also revealed that the proportion of participants with no LTPA participation was higher among those with a low education level (0 to 8 and 9 to 11 years of schooling) in 2019 than among those with a higher education level in 2006, reinforcing the urgency to promote physical activity, especially to socially disadvantaged groups.

In Brazil, the promotion of physical activity within the Unified Health System is part of the national agenda, with the National Health Promotion Policy [30], Family Health Support Centers, and Health Academy Program [31]. The Health Academy Program is free-of-charge, inclusive, and with easy access to people from different socioeconomic backgrounds, as it is located in the same reference territories as the facilities of the Family Health Strategy teams [32]. In 2017, the Health Academy Program was present in 2,678 of the 5,570 Brazilian municipalities, especially in small municipalities with greater social vulnerability [32]. Despite these issues, universal access to the Health Academy Program is not guaranteed, for reasons related to opening hours (only 30% work in the three shifts of the day), lower participation of men, precariousness in the employment relationship of professionals, and some difficulties in articulating with the network of primary health care services, which can lead to interruptions in the provision of activities [33]. Notwithstanding the importance of these policies for physical activity promotion and reduction of inequalities, since 2016, Brazil has undergone fiscal austerity measures that have deepened the underfunding of the Unified Health System and negatively impacted the functioning of these programs.

### Strengths and limitations

Our study has strengths, such as the use of a large sample from 14 different years. On the other hand, some limitations should also be considered, such as physical activity was all self-reported, which could present recall bias. However, currently, there are no viable objective methods available to assess types of physical activity in populational studies. Despite the large population, VIGITEL is representative only of adults living in state capital cities, and consequently, the survey is not representative of non-capital cities and the rural population. The VIGITEL is a telephone-based survey with a sample restricted to people with landline telephones. This limitation in the representativeness could affect the estimates, especially in regions with lower coverage of landline telephones, given that LTPA has been less frequent among those most socially unfavored [34].

Based on our results, strategies to promote physical activity in the population should focus on improving the distribution of physical activity facilities, especially in areas with disadvantaged socioeconomic backgrounds, through the expansion and reinforcement of public policies, such as the Health Academy Program. Furthermore, these strategies could include the improvement of macroeconomic policies to increase the employment rate together with income distribution policies to reduce socioeconomic inequality and the monitoring of barriers to the practice of different types of physical activities among the most disadvantaged groups which, in addition to socioeconomic factors, may include interpersonal factors such as lack of motivation, lack of time, illness and physical limitations [35].

## Conclusion

Even though there was an increase in the participation in different physical activity types, the increase was not equal in all population groups, with women, black and brown people, and subjects with less schooling being the most unfavored groups. In addition, while some inequalities persisted over the years, others increased, such as the ethnicity and education inequalities for strength/gymnastics. Continuous surveillance is essential to explore whether inequalities are reducing, or not and this is even more necessary in the present period, to monitor the inequalities and challenges for physical activity promotion in the “post-COVID era”.

## Supplementary Information

**Additional file 1:** **Table S1.** Trends in the prevalence of different types of LTPA and no LTPA participation among Brazilian adults living in capital cities, according to gender. **Table S2.** Trends in the prevalence ofdifferent types of LTPA and no LTPA participation among Brazilian adults livingin capital cities, according to ethnicity. **TableS3.** Trends in the prevalence of different types of LTPA and no LTPA participation among Brazilian adults living in capital cities,according to education level.

## Data Availability

Data are available under open access through the link: http://svs.aids.gov.br/download/Vigitel/.
