# Supplementary material for: Sociodemographic inequalities in the trends of different types of leisure-time physical activity among Brazilian adults between 2006 and 2019
Source: Int J Equity Health. 2022 Aug 29;21:120. doi: 10.1186/s12939-022-01728-y (PMC9426210; doi:10.1186/s12939-022-01728-y)
Supplement: Supplementary file 1 — Additional file 1: Table S1. Trends in the prevalence of different types of LTPA and no LTPA participation among Brazilian adults living in capital cities, according to gender. Table S2. Trends in the prevalence ofdifferent types of LTPA and no LTPA participation among Brazilian adults livingin capital cities, according to ethnicity. TableS3. Trends in the prevalence of different types of LTPA and no LTPA participation among Brazilian adults living in capital cities,according to education level. [file 12939_2022_1728_MOESM1_ESM.docx]

Table S1 - Trends in the prevalence of different types of LTPA and no LTPA participation among Brazilian adults living in capital cities, according to gender.

|  | Walking | | Running | | Strength/gymnastics | |
| --- | --- | --- | --- | --- | --- | --- |
| Year | Women | Men | Women | Men | Women | Men |
| 2006 | 19.5 (18.2; 20.3) | 15.4 (14.4; 16.4) | 1.0 (0.8; 1.2) | 2.4 (2.1; 2.8) | 9.3 (8.7; 9.9) | 8.3 (7.7; 9.1) |
| 2007 | 21.3 (20.4; 22.1) | 14.2 (13.3; 15.1) | 0.7 (0.5; 0.8) | 3.5 (3.1; 4.0) | 9.0 (8.5; 9.6) | 8.3 (7.6; 9.1) |
| 2008 | 20.1 (19.3; 20.9) | 13.6 (12.8; 14.5) | 0.9 (0.7; 1.1) | 4.1 (3.7; 4.7) | 8.5 (8.0; 9.1) | 7.9 (7.3; 8.6) |
| 2009 | 19.0 (18.2; 19.9) | 14.3 (13.4; 15.2) | 0.9 (0.7; 1.1) | 3.9 (3.4; 4.4) | 9.6 (9.0; 10.2) | 8.4 (7.8; 9.2) |
| 2010 | 18.9 (18.1; 19.8) | 14.1 (13.2; 15.1) | 0.9 (0.7; 1.1) | 4.4 (3.9; 5.0) | 10.0 (9.4; 10.7) | 9.0 (8.3; 9.9) |
| 2011 | 19.2 (18.4; 20.0) | 14.6 (13.8; 15.5) | 1.3 (1.1; 1.6) | 4.6 (4.1; 5.2) | 10.4 (9.8; 11.0) | 10.1 (9.3; 11.0) |
| 2012 | 20.4 (19.4; 21.3) | 15.3 (14.3; 16.3) | 1.6 (1.3; 2.0) | 4.9 (4.3; 5.6) | 12.8 (12.0; 13.6) | 12.0 (11.0; 13.0) |
| 2013 | 19.4 (18.6; 20.3) | 15.0 (14.1; 16.0) | 1.5 (1.3; 1.8) | 4.9 (4.4; 5.6) | 14.6 (13.8; 15.4) | 12.9 (12.0; 13.9) |
| 2014 | 21.2 (20.2; 22.2) | 16.5 (15.4; 17.6) | 1.8 (1.4; 2.2) | 5.3 (4.7; 6.1) | 15.0 (14.1; 15.9) | 12.5 (11.5; 13.7) |
| 2015 | 19.5 (18.5; 20.4) | 16.4 (15.3; 17.6) | 2.0 (1.7; 2.4) | 6.0 (5.3; 6.8) | 18.1 (17.1; 19.0) | 13.4 (12.4; 14.5) |
| 2016 | 20.1 (19.2; 21.0) | 17.6 (16.5; 18.6) | 2.2 (1.9; 2.6) | 6.7 (6.0; 7.5) | 16.3 (15.4; 17.2) | 12.9 (12.0; 13.9) |
| 2017 | 22.1 (21.1; 23.1) | 16.6 (15.5; 17.6) | 2.5 (2.1; 3.0) | 6.8 (6.1; 7.6) | 15.8 (14.9; 16.7) | 13.0 (12.0; 14.1) |
| 2018 | 22.0 (21.0; 22.9) | 17.6 (16.6; 18.7) | 2.4 (2.0; 2.8) | 7.7 (6.9; 8.6) | 17.5 (16.6; 18.4) | 12.3 (11.4; 13.3) |
| 2019 | 22.0 (21.1; 23.0) | 17.7 (16.6; 18.8) | 2.3 (1.9; 2.8) | 6.3 (5.6; 7.1) | 17.5 (16.6; 18.5) | 13.8 (12.8; 15.0) |
| Year | Sports | | Other | | No LTPA practice | |
| 2006 | 2.0 (1.7; 2.3) | 22.4 (21.2; 23.6) | 3.2 (2.9; 3.6) | 5.6 (5.0; 6.3) | 64.7 (63.7; 65.7) | 45.6 (44.2; 47.0) |
| 2007 | 1.8 (1.6; 2.2) | 21.7 (20.5; 22.8) | 3.3 (3.0; 3.7) | 5.3 (4.7; 6.0) | 63.6 (62.6; 64.6) | 46.7 (45.3; 48.0) |
| 2008 | 1.7 (1.4; 2.0) | 20.4 (19.3; 21.5) | 3.9 (3.5; 4.4) | 5.9 (5.2; 6.6) | 64.6 (63.6; 65.6) | 47.7 (46.3; 49.1) |
| 2009 | 1.4 (1.2; 1.7) | 20.8 (19.6; 22.0) | 3.4 (3.0; 3.8) | 6.0 (5.3; 6.7) | 65.4 (64.4; 66.4) | 46.3 (44.9; 47.8) |
| 2010 | 1.3 (1.1; 1.6) | 19.8 (18.7; 21.1) | 3.9 (3.5; 4.3) | 5.3 (4.7; 6.0) | 64.6 (63.6; 65.6) | 47.0 (45.6; 48.5) |
| 2011 | 1.6 (1.3; 1.9) | 20.0 (19.0; 21.0) | 4.6 (4.2; 5.0) | 5.6 (5.1; 6.2) | 62.7 (61.6; 63.7) | 44.7 (43.4; 46.1) |
| 2012 | 1.7 (1.4; 2.1) | 18.4 (17.2; 19.6) | 3.7 (3.3; 4.2) | 5.4 (4.8; 6.2) | 59.5 (58.4; 60.7) | 43.7 (42.2; 45.3) |
| 2013 | 1.7 (1.4; 2.2) | 18.5 (17.4; 19.7) | 3.4 (3.0; 3.8) | 4.7 (4.2; 5.4) | 59.0 (57.9; 60.1) | 43.6 (42.1; 45.1) |
| 2014 | 1.7 (1.4; 2.1) | 18.9 (17.5; 20.4) | 4.3 (3.7; 5.1) | 4.9 (4.2; 5.7) | 55.7 (54.4; 57.0) | 41.5 (39.9; 43.2) |
| 2015 | 2.0 (1.7; 2.5) | 19.2 (17.9; 20.5) | 4.2 (3.8; 4.7) | 5.1 (4.5; 5.9) | 53.8 (52.6; 55.1) | 39.5 (38.0; 41.1) |
| 2016 | 2.6 (2.2; 3.1) | 19.1 (17.9; 20.4) | 5.5 (5.0; 6.1) | 6.6 (5.9; 7.4) | 53.0 (51.8; 54.2) | 36.8 (35.4; 38.2) |
| 2017 | 2.2 (1.9; 2.7) | 18.0 (16.7; 19.3) | 6.0 (5.5; 6.7) | 6.4 (5.7; 7.1) | 51.0 (49.8; 52.2) | 38.9 (37.4; 40.5) |
| 2018 | 2.3 (2.0; 2.7) | 18.5 (17.3; 19.8) | 6.2 (5.6; 6.8) | 6.8 (6.0; 7.7) | 49.5 (48.2; 50.7) | 36.8 (35.3; 38.3) |
| 2019 | 2.4 (2.0; 2.8) | 18.5 (17.2; 19.9) | 6.7 (6.0; 7.4) | 7.1 (6.3; 7.9) | 48.8 (47.5; 50.0) | 36.3 (34.7; 37.8) |

Note: LTPA, leisure-time physical activity.

Table S2 - Trends in the prevalence of different types of LTPA and no LTPA participation among Brazilian adults living in capital cities, according to ethnicity.

|  | Walking | | | | Running | | | |
| --- | --- | --- | --- | --- | --- | --- | --- | --- |
| Year | White | Black | Brown | Yellow/Indigenous | White | Black | Mixed | Yellow/Indigenous |
| 2006 | 19.3 (18.3; 20.4) | 17.4 (15.0; 20.1) | 16.2 (15.4; 17.0) | 21.6 (13.9; 32.1) | 1.9 (1.6; 2.2) | 2.3 (1.5; 3.6) | 1.4 (1.2; 1.6) | 0.5 (0.1; 1.4) |
| 2007 | 20.0 (19.0; 21.0) | 16.9 (14.5; 19.7) | 16.6 (15.8; 17.4) | 19.8 (12.4; 30.0) | 2.1 (1.8; 2.5) | 1.3 (0.8; 2.1) | 2.0 (1.7; 2.4) | 0.07 (0.01; 0.3) |
| 2008 | 19.3 (18.3; 20.3) | 14.9 (12.7; 17.4) | 15.8 (15.1; 16.6) | 14.8 (9.9; 21.6) | 2.8 (2.4; 3.4) | 2.2 (1.5; 3.2) | 2.0 (1.8; 2.4) | 3.7 (0.8; 15.0) |
| 2009 | 18.3 (17.3; 19.3) | 13.4 (11.4; 15.7) | 16.3 (15.4; 17.1) | 19.6 (10.8; 32.9) | 2.3 (2.0; 2.8) | 2.0 (1.2; 3.1) | 2.2 (1.9; 2.6) | 2.3 (0.6; 7.7) |
| 2010 | 19.2 (18.1; 20.2) | 16.0 (13.8; 18.5) | 15.0 (14.3; 15.8) | 9.2 (5.1; 16.0) | 2.7 (2.3; 3.2) | 1.9 (1.3; 2.8) | 2.4 (2.0; 2.9) | 2.4 (0.9; 5.9) |
| 2011 | 18.8 (17.9; 19.8) | 15.6 (13.9; 17.4) | 15.7 (14.9; 16.5) | 16.0 (13.5; 19.0) | 3.2 (2.7; 3.8) | 2.1 (1.5; 2.9) | 2.7 (2.3; 3.2) | 1.8 (1.2; 2.7) |
| 2012 | 19.8 (18.7; 20.9) | 15.0 (13.2; 17.1) | 17.3 (16.3; 18.4) | 15.1 (12.6; 18.0) | 3.5 (3.0; 4.2) | 1.9 (1.3; 2.7) | 3.1 (2.6; 3.7) | 2.5 (1.6; 3.8) |
| 2013 | 18.3 (17.4; 19.3) | 16.4 (14.5; 18.6) | 16.8 (15.8; 17.9) | 15.6 (13.1; 18.6) | 3.6 (3.1; 4.2) | 1.9 (1.5; 2.6) | 2.7 (2.3; 3.2) | 4.8 (2.6; 8.5) |
| 2014 | 20.8 (19.6; 22.0) | 16.1 (14.1; 18.3) | 18.3 (17.1; 19.5) | 16.0 (13.2; 19.2) | 3.2 (2.7; 3.7) | 3.7 (2.6; 5.3) | 3.6 (3.0; 4.2) | 3.2 (1.8; 5.5) |
| 2015 | 18.6 (17.6; 19.6) | 16.3 (13.9; 19.1) | 17.8 (16.6; 19.0) | 18.8 (15.4; 22.7) | 3.8 (3.3; 4.4) | 2.9 (2.1; 3.9) | 4.3 (3.7; 5.1) | 2.6 (1.7; 4.0) |
| 2016 | 19.4 (18.4; 20.4) | 17.5 (15.5; 19.7) | 18.7 (17.7; 19.8) | 19.8 (15.6; 24.7) | 4.3 (3.8; 4.9) | 3.8 (2.8; 5.2) | 4.4 (3.8; 5.1) | 4.6 (2.9; 7.1) |
| 2017 | 20.7 (19.5; 21.8) | 16.6 (14.6; 18.7) | 19.0 (17.9; 20.1) | 21.0 (16.7; 26.0) | 4.2 (3.6; 4.8) | 5.2 (4.0; 6.7) | 4.8 (4.1; 5.5) | 3.8 (2.3; 6.3) |
| 2018 | 20.8 (19.7; 21.9) | 16.6 (14.8; 18.7) | 20.0 (19.0; 21.1) | 19.3 (14.5; 25.1) | 4.3 (3.8; 5.0) | 5.2 (3.9; 6.9) | 5.1 (4.5; 5.8) | 7.2 (3.3; 14.8) |
| 2019 | 20.9 (19.8; 22.0) | 19.3 (17.1; 21.7) | 19.4 (18.3; 20.5) | 20.4 (16.1; 25.5) | 3.9 (3.3; 4.6) | 4.6 (3.5; 6.1) | 4.3 (3.7; 4.9) | 4.6 (2.6; 7.7) |
| Year | Strength/gymnastics | | | | Sports | | | |
| 2006 | 10.3 (9.5; 11.0) | 7.3 (5.7; 9.4) | 7.8 (7.2; 8.4) | 19.3 (10.5; 32.7) | 9.8 (9.0; 10.7) | 13.0 (10.4; 16.1) | 12.6 (11.7; 13.4) | 4.9 (2.5; 9.2) |
| 2007 | 10.6 (9.8; 11.4) | 6.6 (5.3; 8.2) | 7.5 (7.0; 8.1) | 9.1 (4.1; 18.9) | 9.5 (8.7; 10.3) | 14.0 (11.3; 17.1) | 11.8 (11.0; 12.6) | 10.9 (5.4; 20.6) |
| 2008 | 9.7 (9.0; 10.5) | 7.9 (6.2; 9.9) | 7.2 (6.7; 7.7) | 9.90 (5.3; 17.6) | 9.3 (8.5; 10.2) | 11.5 (9.3; 14.1) | 11.0 (10.2; 11.8) | 6.8 (3.2; 13.5) |
| 2009 | 11.1 (10.3; 12.0) | 8.1 (6.2; 10.6) | 7.7 (7.2; 8.2) | 7.1 (3.1; 15.4) | 9.1 (8.2; 10.0) | 11.7 (9.5; 14.4) | 11.1 (10.3; 12.0) | 9.6 (4.1; 21.1) |
| 2010 | 11.0 (10.2; 11.8) | 8.9 (7.1; 11.1) | 8.6 (8.0; 9.3) | 6.9 (2.9; 15.1) | 8.3 (7.5; 9.3) | 10.3 (8.6; 12;4) | 10.9 (10.1; 11.8) | 11.7 (4.6; 26.6) |
| 2011 | 12.2 (11.4; 13.1) | 9.2 (7.8; 10.8) | 8.5 (7.9; 9.2) | 9.8 (7.9; 12.0) | 8.5 (7.8; 9.3) | 13.9 (12.0; 15.9) | 10.7 (9.9; 11.6) | 10.8 (8.5; 13.6) |
| 2012 | 13.6 (12.7; 14.6) | 12.1 (10.1; 14.5) | 11.5 (10.6; 12.5) | 9.8 (7.9; 12.1) | 8.1 (7.3; 9.0) | 10.6 (8.9; 12.7) | 10.1 (9.2; 11.1) | 12.6 (10.0; 15.8) |
| 2013 | 15.2 (14.3; 16.2) | 11.4 (9.7; 13.2) | 13.0 (12.0; 13.9) | 12.7 (10.2; 15.7) | 8.2 (7.4; 9.0) | 11.4 (9.7; 13.4) | 10.4 (9.5; 11.4) | 9.0 (6.3; 12.7) |
| 2014 | 15.4 (14.4; 16.6) | 11.4 (9.6; 13.4) | 12.6 (11.6; 13.7) | 16.3 (12.9; 20.5) | 8.0 (7.1; 9.0) | 12.3 (10.2; 14.9) | 10.2 (9.1; 11.5) | 12.9 (8.9; 18.2) |
| 2015 | 17.8 (16.7; 18.9) | 15.0 (12.2; 18.3) | 14.1 (13.1; 15.1) | 14.8 (12.0; 18.2) | 9.0 (8.1; 10.0) | 12.2 (10.0; 14.9) | 10.6 (9.6; 11.7) | 10.2 (7.7; 13.3) |
| 2016 | 16.2 (15.2; 17.2) | 13.2 (11.3; 15.3) | 13.3 (12.4; 14.3) | 15.0 (11.5; 19.4) | 9.0 (8.1; 9.9) | 14.6 (12.3; 17.2) | 10.6 (9.6; 11.6) | 10.2 (7.3; 14.0) |
| 2017 | 16.0 (15.0; 17.1) | 11.4 (9.7; 13.5) | 13.5 (12.5; 14.6) | 16.8 (12.3; 22.5) | 8.3 (7.4; 9.3) | 12.7 (10.6; 15.2) | 9.9 (8.9; 11.0) | 10.3 (6.9; 15.2) |
| 2018 | 17.5 (16.5; 18.6) | 12.7 (10.7; 15.1) | 13.5 (12.6; 14.4) | 8.3 (6.1; 11.2) | 8.2 (7.3; 9.1) | 15.1 (12.9; 17.7) | 9.8 (8.9; 10.7) | 13.8 (9.1; 20.4) |
| 2019 | 18.5 (17.3; 19.7) | 11.9 (10.1; 14.0) | 14.1 (13.2; 15.1) | 17.0 (12.7; 22.4) | 8.8 (7.8; 9.9) | 13.7 (11.3; 16.5) | 9.8 (8.9; 10.8) | 10.9 (7.8; 15.0) |
| Year | Other | | | | No LTPA practice | | | |
| 2006 | 4.8 (4.2; 5.4) | 3.0 (2.1; 4.3) | 4.1 (3.7; 4.6) | 4.5 (1.8; 10.6) | 53.6 (52.3; 55.0) | 56.7 (53.0; 60.3) | 57.7 (56.5; 58.8) | 48.9 (37.7; 60.2) |
| 2007 | 4.3 (3.8; 4.8) | 5.6 (3.5; 8.8) | 4.1 (3.7; 4.5) | 5.3 (1.6; 16.5) | 53.3 (51.0; 54.7) | 55.3 (51.5; 59.0) | 57.8 (56.6; 58.9) | 54.6 (42.5; 66.1) |
| 2008 | 5.0 (4.5; 5.7) | 4.1 (3.0; 5.6) | 4.7 (4.2; 5.2) | 9.9 (5.3; 17.6) | 53.5 (52.1; 54.8) | 59.1 (55.6; 62.5) | 59.0 (57.9; 60.2) | 51.5 (41.4; 61.4) |
| 2009 | 4.8 (4.2; 5.5) | 3.6 (2.6; 5.0) | 4.5 (4.0; 5.0) | 10.6 (4.9; 21.3) | 54.1 (52.7; 55.5) | 60.9 (57.4; 64.3) | 57.9 (56.7; 59.1) | 50.5 (37.7; 63.2) |
| 2010 | 5.2 (4.6; 5.8) | 3.6 (2.7; 4.9) | 4.1 (3.6; 4.7) | 9.3 (3.7; 21.4) | 53.3 (51.9; 54.7) | 59.0 (55.7; 62.2) | 58.6 (57.4; 59.8) | 60.3 (45.8; 71.4) |
| 2011 | 5.6 (5.1; 6.3) | 4.7 (3.8; 5.9) | 4.5 (4.1; 5.0) | 5.5 (3.9; 7.6) | 51.3 (50.0; 52.6) | 54.2 (51.6; 56.8) | 57.6 (56.3; 58.8) | 55.8 (52.0; 59.6) |
| 2012 | 4.1 (3.6; 4.7) | 3.5 (2.8; 4.5) | 5.2 (4.5; 6.0) | 4.7 (3.4; 6.5) | 50.7 (49.2; 52.1) | 56.6 (53.5; 59.6) | 52.4 (50.9; 53.9) | 55.0 (50.7; 59.2) |
| 2013 | 4.3 (3.8; 4.9) | 3.8 (2.8; 5.1) | 3.6 (3.1; 4.1) | 5.1 (3.4; 7.6) | 50.1 (48.7; 51.4) | 54.8 (52.0; 57.6) | 53.2 (51.8; 54.6) | 52.5 (47.9; 56.9) |
| 2014 | 5.2 (4.4; 6.1) | 3.6 (2.7; 4.8) | 4.4 (3.6; 5.2) | 3.2 (2.2; 4.7) | 47.1 (45.5; 48.7) | 52.6 (49.5; 55.7) | 50.6 (49.0; 52.3) | 48.0 (43.1; 53.0) |
| 2015 | 5.0 (4.4; 5.7) | 4.3 (3.1; 5.8) | 4.4 (3.8; 5.1) | 3.9 (2.8; 5.4) | 45.5 (44.1; 47.0) | 49.0 (45.5; 52.5) | 48.5 (47.0; 50.1) | 49.5 (44.7; 54.3) |
| 2016 | 6.1 (5.5; 6.8) | 5.7 (4.4; 7.2) | 5.8 (5.1; 6.6) | 8.3 (5.6; 12.1) | 44.8 (43.4; 46.1) | 44.9 (42.0; 47.9) | 46.9 (45.4; 48.3) | 41.9 (36.8; 47.1) |
| 2017 | 6.7 (6.0; 7.5) | 4.8 (3.7; 6.2) | 6.1 (5.5; 6.7) | 5.1 (3.4; 7.7) | 43.7 (42.3; 45.2) | 49.0 (46.0; 52.1) | 46.4 (44.9; 47.9) | 42.6 (36.7; 48.9) |
| 2018 | 6.9 (6.1; 7.7) | 5.4 (4.3; 6.9) | 6.2 (5.6; 7.0) | 6.8 (3.0; 14.4) | 42.0 (40.6; 43.5) | 44.5 (41.4; 47.7) | 45.1 (43.7; 46.5) | 44.3 (37.3; 51.6) |
| 2019 | 6.9 (6.2; 7.7) | 5.5 (4.3; 7.0) | 7.2 (6.4; 8.1) | 7.3 (4.9; 10.7) | 40.8 (39.3; 42.2) | 44.7 (41.6; 47.8) | 45.0 (43.5; 46.5) | 39.5 (33.9; 45.5) |

Note: LTPA, leisure-time physical activity.

Table S3- Trends in the prevalence of different types of LTPA and no LTPA participation among Brazilian adults living in capital cities, according to education level.

|  | Walking | | | Running | | | Strength/gymnastics | | |
| --- | --- | --- | --- | --- | --- | --- | --- | --- | --- |
| Year | 0 to 8 | 9 to 11 | 12+ | 0 to 8 | 9 to 11 | 12+ | 0 to 8 | 9 to 11 | 12+ |
| 2006 | 16.3 (15.3; 17.3) | 17.5 (16.6; 18.4) | 20.8 (19.5; 22.1) | 0.7 (0.6; 1.0) | 1.6 (1.3; 2.0) | 3.6 (3.0; 4.3) | 3.9 (3.4; 4.4) | 10.3 (9.5; 11.2) | 17.4 (16.2; 18.7) |
| 2007 | 16.9 (15.9; 17.9) | 17.6 (16.7; 18.6) | 21.1 (19.9; 22.5) | 0.8 (0.5; 1.2) | 2.3 (1.9; 2.7) | 4.2 (3.5; 4.9) | 4.0 (3.5; 4.5) | 10.5 (9.7; 11.3) | 16.4 (15.2; 17.6) |
| 2008 | 16.9 (15.9; 17.9) | 16.7 (15.8; 17.6) | 18.3 (17.2; 19.5) | 1.0 (0.7; 1.3) | 2.5 (2.1; 3.0) | 4.9 (4.2; 5.8) | 3.5 (3.1; 4.0) | 9.4 (8.7; 10.2) | 15.9 (14.8; 17.1) |
| 2009 | 15.4 (14.3; 16.4) | 16.8 (15.9; 17.8) | 19.7 (18.6; 20.9) | 0.8 (0.5; 1.2) | 2.6 (2.2; 3.0) | 4.5 (4.0; 5.2) | 4.1 (3.5; 4.6) | 10.1 (9.3; 11.0) | 16.7 (15.6; 17.9) |
| 2010 | 15.4 (14.3; 16.5) | 16.9 (16.0; 17.8) | 18.9 (17.7; 20.1) | 0.8 (0.5; 1.2) | 2.7 (2.3; 3.2) | 5.1 (4.4; 5.8) | 4.2 (3.7; 4.9) | 10.3 (9.5; 11.2) | 17.6 (16.4; 18.8) |
| 2011 | 16.8 (15.8; 17.9) | 16.3 (15.5; 17.2) | 18.6 (17.5; 19.8) | 1.0 (0.7; 1.4) | 3.0 (2.6; 3.5) | 5.5 (4.8; 6.4) | 5.1 (4.5; 5.8) | 10.6 (9.8; 11.4) | 17.8 (16.6; 19.1) |
| 2012 | 17.3 (16.1; 18.5) | 18.3 (17.3; 19.5) | 18.6 (17.4; 19.9) | 1.2 (0.8; 1.6) | 3.0 (2.5; 3.6) | 6.1 (5.1; 7.2) | 5.8 (5.1; 6.7) | 12.9 (11.9; 13.9) | 21.3 (19.9; 22.8) |
| 2013 | 17.6 (16.3; 18.9) | 17.0 (16.1; 18.0) | 17.7 (16.1; 18.0) | 0.8 (0.6; 1.2) | 3.1 (2.6; 3.6) | 6.0 (5.2; 6.9) | 6.5 (5.7; 7.3) | 13.5 (12.6; 14.5) | 23.3 (21.9; 24.7) |
| 2014 | 19.0 (17.6; 20.4) | 19.9 (18.7; 21.2) | 17.9 (16.6; 19.3) | 1.4 (1.0; 2.0) | 3.5 (2.9; 4.2) | 5.8 (5.0; 6.7) | 6.8 (5.8; 7.8) | 12.9 (11.9; 14.0) | 24.2 (22.5; 25.9) |
| 2015 | 19.1 (17.6; 20.7) | 17.3 (16.2; 18.3) | 17.9 (16.8; 19.1) | 1.3 (0.8; 1.9) | 4.3 (3.7; 5.0) | 6.3 (5.5; 7.1) | 8.2 (7.1; 9.4) | 15.1 (14.0; 16.2) | 25.7 (24.3; 27.1) |
| 2016 | 20.3 (19.0; 21.8) | 18.8 (17.7; 19.9) | 17.7 (16.6; 18.8) | 1.9 (1.4; 2.4) | 4.1 (3.5; 4.8) | 6.9 (6.1; 7.8) | 6.8 (6.0; 7.7) | 12.8 (11.8; 13.8) | 24.4 (23.1; 25.8) |
| 2017 | 20.6 (19.2; 22.0) | 19.9 (18.7; 21.2) | 18.2 (17.0; 19.4) | 1.8 (1.4; 2.5) | 4.8 (4.1; 5.6) | 6.7 (6.0; 7.6) | 6.2 (5.5; 7.0) | 13.2 (12.2; 14.4) | 23.7 (22.3; 25.1) |
| 2018 | 22.7 (21.3; 24.2) | 19.8 (18.7; 20.9) | 17.7 (16.5; 18.9) | 1.4 (1.0; 1.9) | 4.9 (4.3; 5.6) | 7.9 (6.9; 9.0) | 7.0 (6.2; 7.9) | 13.6 (12.6; 14.7) | 24.1 (22.8; 25.5) |
| 2019 | 22.1 (20.6; 23.6) | 19.7 (18.6; 20.9) | 18.6 (17.4; 19.9) | 1.9 (1.4; 2.6) | 4.0 (3.4; 4.8) | 6.3 (5.5; 7.1) | 7.2 (6.3; 8.2) | 13.1 (12.1; 14.2) | 26.1 (24.6; 27.6) |
| Year | Sports | | | Other | | | No LTPA practice | | |
| 2006 | 8.3 (7.5; 9.3) | 15.1 (14.1; 16.2) | 12.1 (11.0; 13.3) | 3.0 (2.6; 3.6) | 4.6 (4.1; 5.3) | 6.5 (5.7; 7.5) | 67.5 (66.1; 68.8) | 50.6 (49.2; 51.9) | 39.2 (37.6; 40.9) |
| 2007 | 8.0 (7.1; 8.9) | 14.7 (13.7; 15.8) | 11.2 (10.1; 12.3) | 2.8 (2.4; 3.4) | 4.9 (4.3; 5.5) | 6.3 (5.5; 7.3) | 67.3 (65.9; 68.6) | 49.7 (48.4; 51.0) | 40.5 (38.9; 42.1) |
| 2008 | 7.0 (6.2; 7.8) | 13.6 (12.6; 14.6) | 11.9 (10.7; 13.1) | 3.8 (3.2; 4.4) | 5.0 (4.4; 5.6) | 6.7 (5.9; 7.7) | 67.6 (66.2; 68.9) | 52.6 (51.3; 53.9) | 41.9 (40.3; 43.5) |
| 2009 | 6.6 (5.8; 7.6) | 14.0 (13.0; 15.1) | 11.5 (10.3; 12.7) | 3.5 (2.9; 4.1) | 4.6 (4.1; 5.1) | 6.7 (5.8; 7.6) | 69.4 (68.0; 70.9) | 51.5 (50.2; 52.9) | 40.6 (39.0; 42.1) |
| 2010 | 6.7 (5.8; 7.8) | 12.9 (11.9; 13.9) | 10.7 (9.6; 11.8) | 3.1 (2.5; 3.7) | 4.6 (4.1; 5.2) | 7.0 (6.1; 7.9) | 69.5 (67.9; 71.0) | 52.3 (51.0; 53.7) | 40.5 (39.0; 42.1) |
| 2011 | 7.1 (6.3; 8.0) | 13.1 (12.2; 14.1) | 10.2 (9.2; 11.3) | 3.3 (2.8; 3.8) | 5.3 (4.8; 5.8) | 7.5 (6.7; 8.4) | 66.4 (65.0; 67.8) | 51.4 (50.1; 52.7) | 40.1 (38.5; 41.7) |
| 2012 | 6.9 (6.0; 8.0) | 11.5 (10.5; 12.5) | 10.0 (8.9; 11.2) | 3.2 (2.6; 3.8) | 4.9 (4.3; 5.6) | 5.9 (5.1; 6.8) | 65.3 (63.7; 66.9) | 49.1 (47.6; 50.6) | 37.9 (36.2; 39.6) |
| 2013 | 5.8 (5.0; 6.8) | 12.5 (11.5; 13.6) | 9.7 (8.8; 10.8) | 3.3 (2.7; 4.0) | 3.7 (3.3; 4.3) | 5.2 (4.6; 5.9) | 65.7 (64.0; 67.3) | 49.9 (48.5; 51.2) | 37.8 (36.2; 39.4) |
| 2014 | 6.4 (5.4; 7.6) | 11.9 (10.7; 13.1) | 10.7 (9.3; 12.3) | 3.4 (2.7; 4.3) | 4.4 (3.7; 5.2) | 6.4 (5.4; 7.5) | 62.7 (60.8; 64.5) | 47.1 (45.4; 48.7) | 34.7 (32.9; 36.6) |
| 2015 | 6.5 (5.5; 7.8) | 12.6 (11.5; 13.8) | 10.5 (9.5; 11.7) | 3.3 (2.7; 4.1) | 4.3 (3.7; 4.9) | 6.6 (5.8; 7.5) | 61.3 (59.3; 63.3) | 46.1 (44.6; 47.6) | 32.7 (31.2; 34.2) |
| 2016 | 6.7 (5.6; 7.9) | 13.2 (12.1; 14.3) | 10.7 (9.7; 11.8) | 4.2 (3.6; 5.0) | 6.3 (5.6; 7.2) | 7.4 (6.6; 8.3) | 59.8 (58.0; 61.6) | 44.4 (42.9; 46.0) | 32.6 (31.2; 34.0) |
| 2017 | 6.4 (5.4; 7.5) | 12.1 (11.0; 13.6) | 9.8 (8.8; 11.0) | 4.5 (3.8; 5.2) | 6.2 (5.6; 7.0) | 7.8 (6.9; 8.8) | 60.3 (58.5; 62.1) | 43.4 (41.8; 45.0) | 33.5 (32.0; 35.1) |
| 2018 | 5.5 (4.5; 6.5) | 12.6 (11.5; 13.7) | 10.6 (9.5; 11.8) | 4.5 (3.7; 5.5) | 6.5 (5.8; 7.3) | 8.1 (7.2; 9.1) | 58.6 (56.8; 60.4) | 42.3 (40.8; 43.8) | 31.3 (29.8; 32.9) |
| 2019 | 6.5 (5.4; 7.9) | 12.5 (11.4; 13.8) | 9.8 (8.7; 11.0) | 4.9 (4.2; 5.8) | 6.8 (6.0; 7.7) | 8.6 (7.6; 9.6) | 57.0 (55.1; 58.9) | 43.5 (42.0; 45.1) | 30.4 (28.9; 31.9) |

Note: LTPA, leisure-time physical activity.
